# Subgroup analysis of the lipid infusion and patient outcomes in sepsis trial (LIPOS) reveals benefit in a subgroup not treated with stress replacement doses of corticosteroids

**DOI:** 10.1186/cc14065

**Published:** 2014-12-03

**Authors:** TS Parker, DM Levine, BR Gordon, SD Saal

**Affiliations:** 1 The Rogosin Institute, New York, NY, USA; 2 Department of Biochemistry, New York Presbyterian Hospital and Weill Cornell Medical College, New York, NY, USA; 3 Department of Medicine, New York Presbyterian Hospital and Weill Cornell Medical College, New York, NY, USA

## Introduction

Lipidose (formerly GR270773) is a protein-free phospholipid emulsion intended for the treatment of hospitalized patients with suspected or confirmed Gram-negative severe sepsis. Lipidose contains phosphatidylcholine, triglyceride and sodium cholate formulated to optimize delivery of the phospholipid component to the surface of high-density lipoprotein (HDL) and other lipoproteins, thereby enhancing the capacity of the patient's circulating lipoprotein pool to bind and neutralize microbial toxins. When Lipidose is infused into blood, the cholic acid is adsorbed onto serum albumin and the phospholipid selectively associates with lipoproteins. Bound and neutralized toxins are removed from the circulation by the liver and excreted along with the cholic acid into the bile. The LIPOS trial enrolled 1,400+ patients at 235 study centers in 31 countries to access Lipidose treatment at two dose levels. The LIPOS headline data presented only a small mortality benefit for the lower dose and no benefit from the higher dose [1]. A subgroup analysis was carried out to test the hypothesis of benefit in the subgroup with adequate liver function, using serum albumin levels as a measure of liver function, and adequate pre-existing HDL or total lipoprotein to accept phospholipid as predicted by the mechanism of action.

## Methods

Albumin, cholesterol and HDL were measured in stored serum samples. The response to treatment and interactions with baseline covariates specified in LIPOS were tested after exclusion of subjects in the lowest biomarker quartiles (AlbTC25 and AlbHDL25).

## Results

Subjects above the lowest quartile of albumin cleared Lipidose significantly faster than those in the lowest quartile (*P *< 0.003). Interactions between treatment and planned use of intravenous stress replacement doses of corticosteroids (IVCST) were found in the AlbTC25 and AlbHDL25 subgroups (*P *< 0.05). Exclusion of these subjects revealed strong relationships between treatment benefit and cholesterol or HDL that were used to select optimal biomarker thresholds. Requiring albumin ≥1.5 g/dl and either cholesterol ≥1 mM or HDL ≥0.5 mM selected 59% and 36%, respectively, of the LIPOS population. Treatment with Lipidose reduced mortality in these subgroups by 6.6% (*P *< 0.025) or 10.8% (*P *< 0.005) respectively. The treatment benefits persisted for at least 1 year.

## Conclusion

A strong negative interaction with IVCST may have masked a significant treatment benefit in LIPOS. This interaction may be related to the ability of bile acids to slow clearance and raise concentrations of corticosteroids [2,3]. Biomarkers can be used to select subjects with early severe septic shock responsive to treatment with Lipidose.
